# I JoY

**DOI:** 10.4103/0973-6131.37570

**Published:** 2008

**Authors:** H R Nagendra

**Affiliations:** Swami Vivekananda Yoga Anusandhana Samsthana (A Yoga University), No.9, Appajappa Agrahara, Chamarajpet, Bangalore - 560 018, India. E-mail: hrn@vyasa.org

“We come to the first issue of the International Journal of Yoga launched on July 16, 2006 by Pujya Swami Dayananda Saraswati, the chair for “Jnana Yoga Peetham” of Swami Vivekananda Yoga Anusandhana Samsthana (SVYASA), a Deemed University. The aforementioned online journal is published by this unique University devoted to Yoga science, combining the best of the East and the West. SVYASA recognized by the Govt. of India, Ministry of Human Resource Development and University Grants Commission in 2002 is part of the nonprofit organization VYASA (Vivekananda Yoga Anusandhana Samsthana) registered as a society in 1986. Over two decades, VYASA has made substantial contributions to yoga research and applications through nearly 70 publications in peer-reviewed journals at national and international levels. With nearly 50 students pursuing their Ph.D. degree and 100 their Master's degree, SVYASA has carried itself as a centre of advanced research in yoga and neurophysiology accredited by the Indian Council of Medical Research (ICMR). Yoga Therapy for Health with a research-based Arogyadhama (wellness home) has treated large numbers of common ailments with fascinating success using yoga therapy as an adjunct to the modern medical system. Thus, it has established the usefulness of the Holistic approach of yoga working at the body, prana, mind, emotional and intellectual levels with a clearly set goal of moving towards perfect health.

Modern medical healthcare delivery systems have made tremendous strides in alleviating infectious and contagious diseases as well as the pain, agony and distress of these ailments. However, the exacting scientific rigor of their body-based mechanistic approach has failed to deal with the multidimensional challenge of ailments as asthma, Diabetes Mellitus, coronary artery diseases, cancer etc. Specialization and superspecialization, which became a necessity in this matter-based paradigm led to greater fragmentation but also resulted in ‘no cure’ situations while trying to combat the pandemic of the new era of science and technology. Escalating costs of treatment in modern medicine with no cure and secondary offshoots have made the public move towards alternate complementary and traditional systems of medicine against the advice of modern medical experts. Among CAM and traditional medical systems, applications of yoga have started meeting the needs of evidence-based systems with increasing popularity in India and throughout the world.

While yoga is practiced as yogic postures in thousands of centers world wide, therapeutic yoga is hardly used by authentic personnel of healthcare delivery systems. While most CAM have been looking at naturopathy, acupuncture, herbal medicine etc, SVYASA has started this unique research online journal in the field of yoga and its applications under the following five main heads:

Yoga—Spirituality

Yoga and Life Sciences

Yoga and Physical Sciences

Yoga and Management Studies (including education)

Yoga and Humanities (including sports, fine arts, etc)

This journal being edited by an expert team and peer-reviewed by an International Advisory Board containing experts from all fields of yoga applications, would serve as a focal point for contributions from yoga researchers in its widest sense.

We invite all levels of yoga personnel to contribute their research findings in this unique online journal so as to not only unearth secrets from ancient texts of yoga but also include modern scientific research articles.

